# Supplementary material for: A qualitative evidence synthesis exploring people after stroke, family members, carers and healthcare professionals’ experiences of early supported discharge (ESD) after stroke
Source: PLoS One. 2023 Feb 13;18(2):e0281583. doi: 10.1371/journal.pone.0281583 (PMC9925006; doi:10.1371/journal.pone.0281583)
Supplement: S1 File — (DOCX) [file pone.0281583.s001.docx]

**S1 File. Search String**

| S1 | Acute stroke OR stroke OR Cerebrovascular accident OR CVA* OR Cerebrovascular disorders OR infarct OR incident stroke brain injury, chronic OR post stroke OR poststroke OR post-stroke OR cerebrovasc* |
| --- | --- |
| S2 | Qualitative OR qualitative research OR experience OR experiences OR perception OR perceptions OR perspective OR perspectives OR lived experience OR interview* OR focus group* OR ethnograph* OR phenomenol* OR grounded theor* OR grounded-theor* OR narrative analysis OR ethnological research OR ethnomethodology* |
| S3 | Early supported discharge OR early discharge service* OR Early supported hospital discharge OR ESD OR early discharge OR early OR earlier OR rapid discharge OR prompt OR accelerate* discharge OR acute OR subacute OR supported discharge OR transition of care OR Rehabilitation OR rehab OR rehabilitate OR home rehabilitation OR home therapy OR Patient discharge OR progressive patient care OR discharge OR home care services |
| S4 | S1 + S2 + S3 |
